# Should commercial sex workers have unrestricted healthcare access across the world?

**DOI:** 10.1186/s12939-021-01575-3

**Published:** 2021-10-30

**Authors:** Simon D. Taylor-Robinson, Paulo A. De Souza Lopes, Jey Zdravkov, Rachel Harrison

**Affiliations:** 1 Department of Surgery and Cancer, Imperial College London, St Mary’s Hospital Campus, London, W2 1NY UK; 2 Department of Medicine, UAI Universidad Abierta Interamericana, C1147 AAH Buenos Aires, Argentina; 3 Dean St Sexual Health Clinic, 56 Dean Street, Soho, London, W1D 6AE UK; 4 Department of South East Asian Studies, School of Oriental and African Studies, London, WC1H 0XG UK

**Keywords:** Sex workers, Decriminalization, Healthcare, Human rights, COVID-19

## Abstract

We argue commercial sex workers have rights to healthcare and psychosocial support. While decriminalization is not legally enacted in most countries, we would suggest these workers rights include freedom from harassment and opportunities to lead healthy lives. The need for healthcare access for all is heightened in the COVID-19 pandemic where some people flout rules on lockdown by engaging with commercial sex workers and may unwittingly spread SARS-CoV-2 in so doing. Unrestricted healthcare access without stigma for commercial sex workers protects them, and has a beneficial societal effect on those who engage with them and on their contacts.

## Introduction

Human society is diverse sociologically. Whether it has been ancient Sumeria, mediæval Italy or current day Amazon tribes, all have been united by a code of rules [1]. Society needs the majority of people to be compliant for good governance. Equally well, all societies have codes to punish rule breakers, whether it be human sacrifices of the Aztecs or incarceration in Gulag hard labour camps of Soviet Russia to modern Declarations of Helsinki [1].

Most recent or current societies deem stable family relationships as being the norm, but despite this, all have had to take on or reject prostitution of some form from Biblical Sodom and Gomorrah to the concubines of Imperial China (we now term prostitution “commercial sex work” to avoid negative historical connotations) [2].

Whether overt or covert, commercial sex work exists in all modern day societies in differing forms. While many engage in this work happily through choice and because it may be financially rewarding, there are still some people in certain parts of the world where choice is not necessarily an option [3].

Worldwide, there are estimated to be upwards of 40 million commercial sex workers [4]. It is thought that 80% of such commercial sex workers in the world are women, ranging in age from 13 years to 25 years [4]. It is also estimated that there are in excess of 8.5 million men who are commercial sex workers [4]. Age, sex, ethnicity, immigration status, economic and educational status vary from country to country with poorer and less educated people in the countries of the Global South and more economically advanced people in countries, such as the Netherlands and Norway [4].

Healthcare access for commercial sex workers also varies from country to country with fewer rights of access in countries where commercial sex work carries more severe penal action and easier access in countries of Northern Europe, such as the United Kingdom, where healthcare is free at the point of care for all individuals [4].

While we cannot highlight the situation of commercial sex workers in every country in the world, we have picked a few examples highlighted by Amnesty International [3]. In Papua New Guinea, a good example of a low or middle income country, commercial sex workers have been subject to violence, including by the police [5]. They have experienced considerable stigma and discrimination in their communities, particularly when seeking healthcare for both psychological support and physical ailments, including treatment for sexually transmitted diseases, HIV and also emergency care, when suffering physical violence [5]. This therefore leads to fear of seeking medical treatment in case they are reported to the legal authorities.

In Hong Kong, selling sex is not currently illegal, if the commercial sex worker works alone in a private apartment [6]. This can be a vulnerable situation, often associated with physical attack, which has included abuse by the police when reporting such crimes [6]. Again, fear of being exposed to the authorities may limit healthcare access.

Argentina is an example of a country that ostensibly has a more liberal attitude to commercial sex workers [7]. In Buenos Aires, selling or buying sex by is not formally against the law, but in reality, such practices are criminalized by a series of laws, punishing the methods of communication involved in buying or selling sex, in addition to anti-trafficking laws that do not distinguish between commercial sex work and human trafficking into the sex sector [7]. Again, access to healthcare may be limited owing to fear of reprisal if medical advice is sought.

It is extremely important not to conflate commercial sex work with human trafficking or indeed, with criminal activity of any form, but there are still individuals who perform this work who may have been involved in slavery in some countries of the African Sahel or who are trafficked largely unwillingly in a broad swathe of the world from the Hill Tribes of South East Asia through to those indentured by poverty in South-East Europe [8].

A report by the United Nations Office on Drugs and Crime estimated in 2010 that sexual exploitation was the underlying reason for around 80% of those identified as being subject to human trafficking [9]. The extent of this trade in human lives is almost impossible to accurately assess because commercial sex work is illegal in most countries. It is therefore difficult to distinguish the extent of coerced commercial sex work from voluntary commercial sex work in the majority of countries around the world [6, 7]. However, according to the United States State Department in 2008, 600,000 to 800,000 people were trafficked across national borders, and of these 80% were female with up to 50% being under age [10]. The vulnerable nature of such people highlights the need for the medical profession to be mindful of the problems faced by commercial sex workers and the need to provide a welcoming healthcare environment free from perceived stigma or discrimination.

We suggest in this article that as all present day societies have commercial sex workers, there is a societal duty to protect these people from abuse, and also to provide them with appropriate healthcare access when needed, not only for their benefit, but also for the benefit of those who engage with them and for society at large [5]. We believe that this need for healthcare equality is heightened in the COVID-19 epidemic, where rules on lockdown have at times been flouted. Fully-protected commercial sex workers should not present a significant transmission risk to the community at large in the COVID-19 pandemic if they gain appropriate access to medical services, including COVID-19 vaccination.

### Models of social control

Throughout large parts of the world, commercial sex workers operate illegally with threat of imprisonment or police harassment being real issues. This worldwide problem is highlighted by a campaign from Amnesty International which began in 2016 [3].

However, there are differing models of social control with respect to commercial sex workers enacted into legislation. These laws vary from Nordic countries, where it is illegal to buy sex, but not to sell the use of one’s own body (Sweden), through various local governmental regulatory models (The Netherlands, Germany, Austria) to decriminalization (New Zealand and specific states of Australia) [11, 12]. While The Netherlands has not decriminalized commercial sex work, it has a regulatory model that brings it within the surveillance of local municipal agencies. Given that caveat, adult commercial sex workers in The Netherlands are relatively fortunate, compared to those in the Global South [11]. They may earn a taxable, but pensionable wage, have access to social security and do not have to worry about police brutality, because society for the most part, has recognised that they perform a function for the oppressed, confused, psychologically disturbed or even just the curious who consciously choose to engage their services [11]. Despite continuing social stigma, many enter this work in Netherlands of free choice and some enjoy what they do as a social service [12].

In contrast to the streets of Amsterdam, we would argue that most commercial sex workers around the world are vulnerable, often underage and need to be protected [12]. Legislation will never stamp out such work, as it still occurs even in the strictest of regimes today [11]. What is needed is compassion for commercial sex workers to improve their healthcare, their day-to-day lives and to have understanding, without moral judgment about what they do, if they do so willingly.

### Associated criminal activity

What can propagate large scale commercial sex work is association with criminal activity. In some parts of the world, such work can be a cover for drug running, human trafficking and organised crime [13]. The pattern varies in different areas of the world with Africa and East Asia associated more more human trafficking and the Americas more with drug running [14]. Commercial sex workers who may be caught up in such criminal activity are often targets of police attention [3]. We believe that they are people who require help and compassion, rather than being made guilty of crimes of association.

Often underage girls and boys, frequently indentured in South-East Asia, need support with stigma that haunts their lives, even when they cease to perform this work [15]. Most have no access to medical or psychosocial support as they are marginalized by the societies that bore them, and whose social structures are usually complicit in producing the “need” for their services in the first place [16].

In order to address this, countries, such as New Zealand, have decriminalized commercial sex work in the hope that the associated crime that has accompanied it can be quelled and that those who are involved can be protected from those who wish to take advantage of them [17]. While decriminalisation may possibly help to reduce the associated crime that can accompany commercial sex work, healthcare inequality still remains a reality, most often because of the perceived potential of discrimination [6, 7].

### Healthcare inequality

Commercial sex work is deemed to be sexual misconduct by many societies and on statute books, it often may be considered deviant and punishable [3]. In the current climate, some commercial sex workers are economically or socially oppressed, or indentured [15]. Seeking healthcare can lead to imprisonment or even capital punishment in some countries of the Global South [3, 15].

As has been highlighted in the Amnesty International report, commercial sex workers are of particular concern, as they are vulnerable to abuse from their clients, from the police and from members of their communities around the world, leading to healthcare avoidance, for fear of physical or legal retribution [3]. In the COVID-19 pandemic, most have not been vaccinated, owing to poor access to healthcare; and through close contact with their clients, they are susceptible to contracting the virus and/or spreading it. We believe commercial sex-workers deserve full access to healthcare, including COVID-19 vaccination, and the basic human rights meted out to all in society, because for the most part, these workers are casualties, either physically, economically or psychologically of systems beyond their control and of societies who create a demand for their services [15].

In some areas of healthcare access, commercial sex workers are not unique. Where these individuals are illegal immigrants (as may be the case in the United States, for example), improving access to healthcare for all illegal immigrants (undocumented people) would be beneficial to the whole community, including the sex workers [18].

However, we believe all societies should do more to help commercial sex workers from a psychosocial, educational and healthcare point of view [8]. The plight of the male commercial sex workers of the both the eastern and western African littoral who service Scandinavian, German and Italian tourists is of concern, for they are invisible in their societies and unable to access healthcare [19]. In addition to the stigma of commercial sex work, having same-sex relations is often illegal in sub-Saharan Africa and may also carry threats of life imprisonment or the death penalty, so many are unable to access HIV medication or PrEP for fear of societal retribution [20].

Economic circumstance may drive these young men to become commercial sex workers, as tourism from the Global North feeds the demand. However, nobody should die of HIV or viral hepatitis because of the fear of accessing healthcare. More should be done to highlight the problems of sex tourism for the African victims, rather than hiding the issue under the carpet and pretending that it does not exist.

By contrast, in Thailand, much of the commercial commercial sex work is not originally rooted in the tourist industry at all, but has traditionally served a local market with a deep-rooted history of social-sexual relations [21]. This is likely the case in other countries as well. Nevertheless, commercial sex workers in these countries also need healthcare equality, as societal stigma that this work carries is often life-long.

From a medical standpoint, offering welcoming access to healthcare without judgement, such as in the Praed Street Project in London, United Kingdom, is an area of best practice [22].

The Praed Street Project provides healthcare services for sex workers and has been hailed as a model for other healthcare centres, both within the British National Health Service (NHS) and abroad [22]. The Project is based at St Mary’s Hospital in the Paddington district of London (London Borough of Westminster) and provides a comprehensive series of services to commercial sex workers, with telephone or clinic appointments, or contact through community outreach, including:

- Vaccinations against hepatitis A and B and human papilloma virus (HPV).
- Free condoms and for women: contraception, including the “pill”, depot injection (Depo-Provera) and contraceptive implants.
- Free emergency contraception for women (available 72 h following unprotected sex).
- Advice on PrEP and post-exposure prophylaxis (PEP) with emergency walk-in clinic access if medications are needed.
- Appropriate advice on sexual health at work and home, and information about infections and how to prevent them.
- Answers to questions about safer sex, broken condoms and other issues of concern.
- Information on condom use.
- Confidential testing and regular check-ups for sexually transmitted infections (STIs) including HIV (counselling is also offered with HIV testing).
- Cervical smears
- Medical appointments for advice on other health issues.

While most sex workers do not see themselves as victims, as it may be of free choice, particularly in the Global North, societal stigma may still abound, meaning access to free or assisted healthcare may be limited; in many countries, there is no easy access to PrEP [12, 20]. Seeking it out may be dangerous in terms of legal and judicial penalties in a large number of countries of the Global South.

## Conclusion

The main barrier to healthcare for most commercial sex workers around the world is perceived stigma and discrimination and the potential threat of legal consequences if healthcare is sought [6, 7]. We therefore believe that healthcare providers should be reminded to reflect on their own biases, unconscious or otherwise, when assessing who deserves healthcare. Healthcare systems must reframe the ways in which they have systematically oppressed specific populations of people and modify their structure accordingly. Reducing healthcare inequality has direct benefits for commercial sex workers and for those who court their services, not only in terms of reducing HIV transmission and of sexually transmitted infections, but in the COVID-19 pandemic, reducing potential SARS-CoV-2 virus transmission if such workers are aware of their viral status. Societies generate the demand for commercial sex workers in the first place and we would argue that they should be cognisant of the benefit of healthcare equality, not only for commercial sex workers, but by implication, for society at large.

While decriminalization of commercial sex work is a distant thought for most countries, the medical profession should look to ensure that such commercial sex workers are not marginalized as far as their medical needs are concerned, while the debate continues on whether decriminalization is acceptable in countries around the world. Access to healthcare is a human right for all and is enshrined in Article 25 of the United Nations Declaration of Human Rights [23]. The rights of all individuals, including commercial sex workers should be viewed in this context, ensuring protection without fear, above and beyond criminality when accessing healthcare at any and every point of need [23].

In this context, the UN Declaration of Human Rights has also stated that the right to healthcare should be legally enforceable:

“*Every human being is entitled to the enjoyment of the highest attainable standard of health, conducive to living a life in dignity. The realization of the right to health may be pursued through numerous, complementary approaches, such as the formulation of health policies, or the implementation of health programmes developed by the World Health Organization (WHO), or the adoption of specific legal instruments. Moreover, the right to health includes certain components which are legally enforceable*.”[23, 24]

Furthermore, the UN has declared that Governments around the world need to take all necessary measures to safeguard all people within their jurisdiction from infringements of the right to health, including protecting consumers and workers from practices detrimental to health, protecting women against violence and to prosecute perpetrators of such violence; and to discourage the continued observance of harmful traditional medical or cultural practices. Failure to do so would be at variance with international law [24].

We therefore believe that commercial sex workers, in line with all vulnerable or marginalised groups are entitled to equality of healthcare access under international law as a human right and that governments should be mindful of their obligations to all their citizens [23, 24].

## Data Availability

Not applicable.
